# Removal of Moles by Electricity

**Published:** 1890-02

**Authors:** 


### REMOVAL OF MOLES BY ELECTRICITY.

If the growth is the elevated, warty mole, its removal by electricity is admirable. The brown pigment mole may also be destroyed in this way, but it is not quite as clean-cut an operation. With the warty mole I inject into it a few drops of four or six per cent. cocaine, and as soon as it is benumbed I transfix the growth at the base, exactly on the level at the adjacent skin, the needle having previously been connected to the negative pole of a galvanic battery. I use about twenty cells of a thirty-six-cell battery, if in good condition. One could use from twelve to sixteen, or even twenty, cells. The positive sponge electrode may be held by the patient in the hand, unless the current would pass through the eye, when a small sponge electrode must be pressed upon the face at some point that would avoid this objection.

Very soon after the current is completed bubbles of gas pass out along the points of entrance and exit of the needle, and the mass becomes blanched. Hold the needle there half a minute to a minute. Transfix again at right angles, taking care to interrupt the current by taking off the sponge electrode before the needle is withdrawn.—*Medical News.*

